# Distribution of the *C9orf72* hexanucleotide repeat expansion in healthy subjects: a multicenter study promoted by the Italian IRCCS network of neuroscience and neurorehabilitation

**DOI:** 10.3389/fneur.2024.1284459

**Published:** 2024-01-31

**Authors:** Emiliano Giardina, Paola Mandich, Roberta Ghidoni, Nicola Ticozzi, Giacomina Rossi, Chiara Fenoglio, Francesco Danilo Tiziano, Federica Esposito, Sabina Capellari, Benedetta Nacmias, Rossana Mineri, Rosa Campopiano, Luana Di Pilla, Federica Sammarone, Stefania Zampatti, Cristina Peconi, Flavio De Angelis, Ilaria Palmieri, Caterina Galandra, Eleonora Nicodemo, Paola Origone, Fabio Gotta, Clarissa Ponti, Roland Nicsanu, Luisa Benussi, Silvia Peverelli, Antonia Ratti, Martina Ricci, Giuseppe Di Fede, Stefania Magri, Maria Serpente, Serena Lattante, Teuta Domi, Paola Carrera, Elisa Saltimbanco, Silvia Bagnoli, Assunta Ingannato, Alberto Albanese, Fabrizio Tagliavini, Raffaele Lodi, Carlo Caltagirone, Stefano Gambardella, Enza Maria Valente, Vincenzo Silani

**Affiliations:** ^1^Genomic Medicine Laboratory UILDM, IRCCS Santa Lucia Foundation, Rome, Italy; ^2^Department of Biomedicine and Prevention, Tor Vergata University, Rome, Italy; ^3^IRCCS Ospedale Policlinico San Martino – UOC Genetica Medica, Genova, Italy; ^4^Department of Neuroscience, Rehabilitation, Ophthalmology, Genetics and Maternal and Child Health, University of Genova, Genova, Italy; ^5^Molecular Markers Laboratory, IRCCS Istituto Centro San Giovanni di Dio Fatebenefratelli, Brescia, Italy; ^6^Department of Neurology and Laboratory of Neuroscience, IRCCS Istituto Auxologico Italiano, Milan, Italy; ^7^Department of Pathophysiology and Transplantation, “Dino Ferrari” Center, Università degli Studi di Milano, Milan, Italy; ^8^Unit of Neurology V – Neuropathology, Fondazione IRCCS Istituto Neurologico Carlo Besta, Milan, Italy; ^9^Fondazione IRCCS Ca' Granda, Ospedale Maggiore Policlinico, Milan, Italy; ^10^Department of Biomedical, Surgical and Dental Sciences, University of Milan, Milan, Italy; ^11^Section of Genomic Medicine, Department of Life Sciences and Public Health, Università Cattolica del Sacro Cuore, Rome, Italy; ^12^Unit of Medical Genetics, Department of Laboratory Science and Infectious Diseases, Fondazione Policlinico Universitario A. Gemelli IRCCS, Rome, Italy; ^13^Neurology Unit, IRCCS San Raffaele Scientific Institute, Milan, Italy; ^14^Laboratory of Human Genetics of Neurological Disorders, Division of Neuroscience, Institute of Experimental Neurology, IRCCS San Raffaele Scientific Institute, Milan, Italy; ^15^IRCCS Istituto delle Scienze Neurologiche di Bologna, Bologna, Italy; ^16^DIBINEM Università di Bologna, Bologna, Italy; ^17^Department of Neuroscience, Psychology, Drug Research and Child Health (NEUROFARBA), University of Florence, Florence, Italy; ^18^IRCCS Fondazione Don Carlo Gnocchi, Florence, Italy; ^19^Laboratory Medicine, Department of Cytogenetics and Molecular Genetics, IRCCS Humanitas Research Hospital, Milan, Italy; ^20^IRCCS Neuromed, Pozzilli, Italy; ^21^Department of Mental, Physical Health and Preventive Medicine, University of Campania Luigi Vanvitelli, Naples, Italy; ^22^Department of Biology, California State University, Northridge, Northridge, CA, United States; ^23^IRCCS Mondino Foundation, Pavia, Italy; ^24^Department of Molecular Medicine, University of Pavia, Pavia, Italy; ^25^Department of Medical Biotechnology and Molecular Medicine, Università degli Studi di Milano, Milan, Italy; ^26^Unit of Medical Genetics and Neurogenetics Fondazione IRCCS Istituto Neurologico Carlo Besta, Milan, Italy; ^27^Department of Biological and Environmental Sciences and Technologies, University of Salento, Lecce, Italy; ^28^Experimental Neuropathology Unit, Division of Neuroscience, Institute of Experimental Neurology, IRCCS San Raffaele Scientific Institute, Milan, Italy; ^29^Laboratory of Clinical Molecular Biology, Unit of Genomics for Human Disease Diagnosis, Division of Genetics and Cell Biology, IRCCS San Raffaele Scientific Institute, Milan, Italy; ^30^Department of Neurology, IRCCS Humanitas Research Hospital, Rozzano, Milan, Italy; ^31^Fondazione IRCCS Istituto Neurologico Carlo Besta, Milan, Italy; ^32^Policlinico S. Orsola-Malpighi, Department of Biomedical and NeuroMotor Sciences (DiBiNeM), University of Bologna, Bologna, Italy; ^33^Department of Clinical and Behavioral Neurology, IRCCS Fondazione Santa Lucia, Rome, Italy; ^34^Department of Biomolecular Sciences, University of Urbino "Carlo Bo", Urbino, Italy

**Keywords:** *C9orf72*, GGGGCC hexanucleotide repeat, amyotrophic lateral sclerosis, frontotemporal dementia, allele distribution

## Abstract

**Introduction:**

High repeat expansion (HRE) alleles in *C9orf72* have been linked to both amyotrophic lateral sclerosis (ALS) and frontotemporal dementia (FTD); ranges for intermediate allelic expansions have not been defined yet, and clinical interpretation of molecular data lacks a defined genotype–phenotype association. In this study, we provide results from a large multicenter epidemiological study reporting the distribution of *C9orf72* repeats in healthy elderly from the Italian population.

**Methods:**

A total of 967 samples were collected from neurologically evaluated healthy individuals over 70 years of age in the 13 institutes participating in the RIN (IRCCS Network of Neuroscience and Neurorehabilitation) based in Italy. All samples were genotyped using the AmplideXPCR/CE *C9orf72* Kit (Asuragen, Inc.), using standardized protocols that have been validated through blind proficiency testing.

**Results:**

All samples carried hexanucleotide G_4_C_2_ expansion alleles in the normal range. All samples were characterized by alleles with less than 25 repeats. In particular, 93.7% of samples showed a number of repeats ≤10, 99.9% ≤20 repeats, and 100% ≤25 repeats.

**Conclusion:**

This study describes the distribution of hexanucleotide G_4_C_2_ expansion alleles in an Italian healthy population, providing a definition of alleles associated with the neurological healthy phenotype. Moreover, this study provides an effective model of federation between institutes, highlighting the importance of sharing genomic data and standardizing analysis techniques, promoting translational research. Data derived from the study may improve genetic counseling and future studies on ALS/FTD.

## 1 Introduction

Amyotrophic lateral sclerosis (ALS) is a fatal neurodegenerative disorder characterized by motor impairment with progressive paralysis and cognitive and/or behavioral changes. Death typically occurs in approximately 3 years from the onset of respiratory insufficiency. Familial forms account for approximately 10% of cases, showing an autosomal dominant with reduced penetrance transmission pattern, while the remaining cases are sporadic. Yet, approximately 10% of patients affected by apparently sporadic ALS carry a mutation in one of the genes associated with familial ALS (*SOD1*, *FUS*, *TDP-43,* and *C9orf72*) (1, 2). Frontotemporal dementia (FTD) is a group of neurodegenerative diseases characterized by the progressive impairment of behavior, language, and cognitive functions. The typical onset occurs before 65 years of age, with death within 7 years. A positive family history is described in approximately 25–30% of cases, and the frequency of genetic mutations involved in the inheritance of FTD is different across populations (3). Scientific research in the last decades has improved the genetic characterization of both ALS and FTD. Clinical studies supported by neuropathological and genetic evidence have demonstrated the etiopathological continuum between these disorders, sharing pathogenic mechanisms and common genetic signatures. In 2011, two independent studies (4, 5) identified the hexanucleotide G_4_C_2_ expansion in the non-coding region between exons 1a and 1b of the *C9orf72* gene as the molecular key player of the FTD/ALS complex phenotype (6). Subsequently, several studies confirmed this association, revealing that carriers of a high-repeat expansion (HRE) allele in *C9orf72* develop ALS and/or FTD with variable clinical expression and age-dependent penetrance (7).

Genotyping of the hexanucleotide repeat expansion in *C9orf72* is recommended in patients with a positive family history of ALS, FTD, or both (7). Interestingly, the revaluation of sporadic ALS and FTD patients demonstrated that many subjects carried a hexanucleotide repeat expansion in *C9orf72* (1, 4, 5). In this scenario, it is possible that many familial cases are still unrecognized, maybe for Gompertzian inter-disease competition (8) or other environmental and clinical conditions. In fact, it is well known that approximately 35% of *C9orf72* patients have an atypical presentation mimicking other neurodegenerative disorders (Parkinson’s disease, Huntington’s disease, Lewy body dementia, Alzheimer’s disease, and parkinsonism) that can lead to misdiagnosis (3, 9). It is noteworthy that in certain European populations, the hexanucleotide repeat expansion in *C9orf72* has been identified as the most prevalent cause of phenocopies resembling Huntington’s disease (HD) (10). In line with this, the German Neurological Society has officially recognized *C9orf72* expansion alleles as a primary HD phenocopy in their guidelines for the differential diagnosis of chorea (11). Furthermore, the phenotypes associated with *C9orf72* can vary significantly, even within the same family lineage, manifesting in diverse neurodegenerative presentations (12).

Behind the clinical difficulties in the recognition of *C9orf72-associated* phenotypes, a lack of definition of hexanucleotide repeat cutoff ranges further complicates the diagnosis and interpretation of genotypes. As it stands, there is no universally shared consensus defining the thresholds for normal and pathological *C9orf72* alleles. Various research studies and commercial laboratories have presented differing reference ranges for normal, intermediate, and expanded/pathological *C9orf72* hexanucleotide alleles (13, 14). The range for normal alleles varies from fewer than 20 to fewer than 30 repeat units (7), while pathological alleles are reported to range from over 23 to more than 45 repeat units (7, 15, 16). This variance leads to a scenario where an individual with a *C9orf72* allele containing between 23 and 30 repeats could be classified as carrying either a wild-type or a pathological allele, depending on the laboratory’s adopted threshold. This lack of standardization in *C9orf72* testing thresholds may lead to significant confusion and misinterpretation. Moreover, even in the era of next-generation sequencing, accurately sizing and interpreting repetitive genetic variants remains a significant challenge. This complexity is often due to the reliance on less common analytical methods, many of which are based on homemade protocols and vary considerably across different diagnostic centers. While the concept of method harmonization is straightforward and necessary, practical experience underscores numerous challenges in standardizing technological, methodological, and interpretative steps across these centers. As a result, different analytical methods are used to estimate expanded G_4_C_2_ repeats, often with limited confidence (13, 14). This uncertainty is exacerbated by factors such as the high GC content, large size, somatic instability, repetitive nature of the flanking sequences, and the presence of sequence variations at the 3′ end of the region (17, 18).

**Figure 1:**
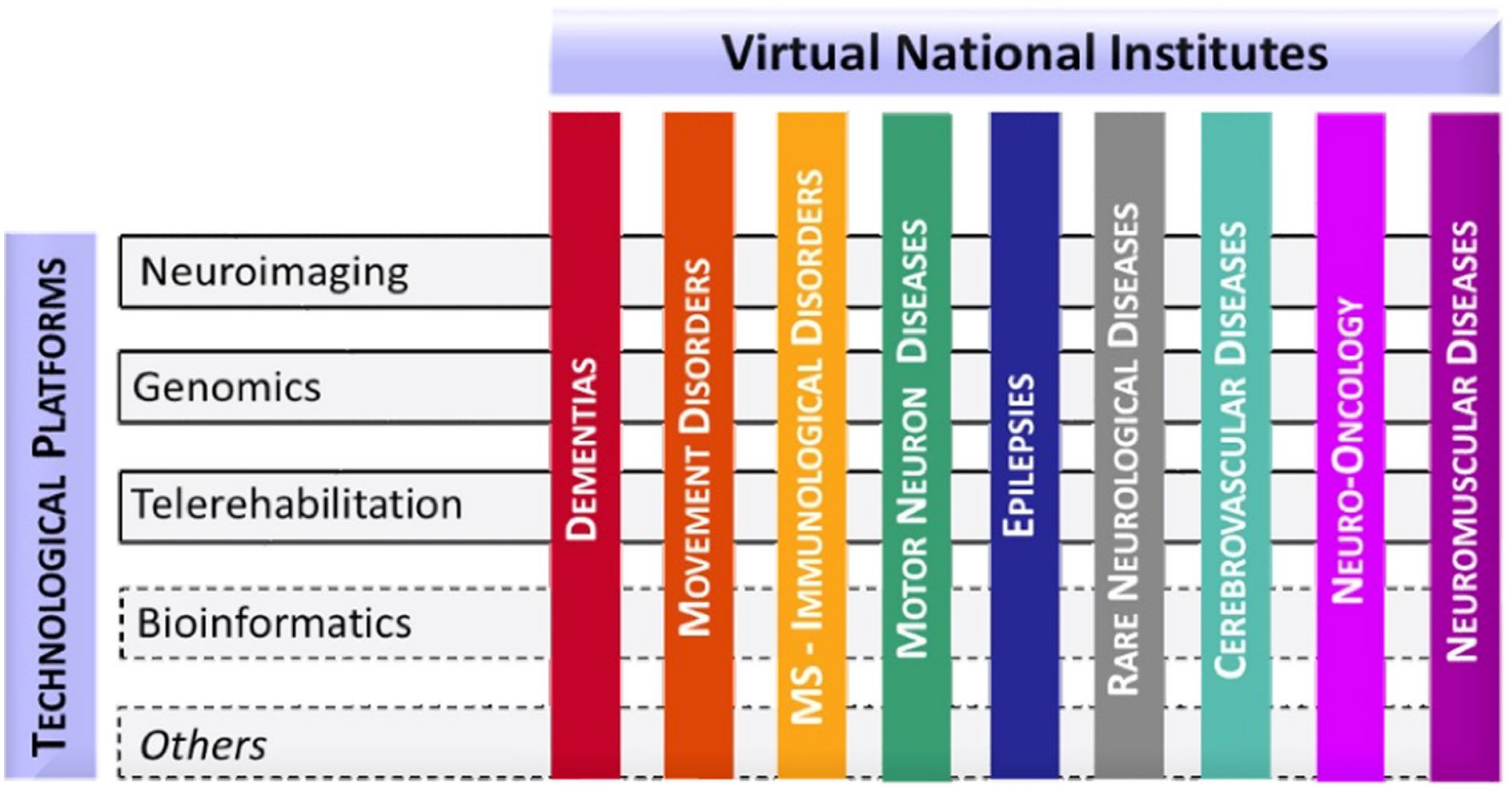
Organizational model of RIN.

In an effort to standardize the genetic testing and interpretation of results for *C9orf72*, the Italian IRCCS Network of Neuroscience and Neurorehabilitation (RIN) conducted a multicenter study. RIN, being the largest federation of Scientific Institutes for Research, Hospitalization, and Healthcare (IRCCS) in Italy with a focus on neuroscience, offers nationwide access to medical genetic data for translational research in compliance with the EU - General Data Protection Regulation. Established in 2017 by the Italian Ministry of Health, RIN aims to foster collaboration among IRCCS centers, facilitate the sharing of clinical-scientific data, and coordinate the development of protocols and algorithms for translational purposes. The network supports scientific and technological research in the prevention, diagnosis, treatment, and rehabilitation of neurodegenerative disorders, including neurological, neuropsychiatric, and related conditions. Given the wide range of neurological phenotypes, effective research requires segmentation into specific thematic areas. To this end, RIN has initiated Virtual Institutes of Pathology (VIP), each dedicated to particular diseases or disease groups (such as dementias, movement disorders, immunological disorders, motor neuron diseases, epilepsies, cerebrovascular disorders, neuro-oncology, and rare neurological disorders). RIN’s structure comprises these VIPs, involving diagnostic and research centers active in patient management and cross-disciplinary technological platforms (including neuroimaging, genomics telerehabilitation, and bioinformatics) for centralized and standardized analyses (as illustrated in Figure 1). This study presents the outcomes of this national endeavor to harmonize and standardize the typing of *C9orf72* expansions. Additionally, we detail the distribution of hexanucleotide G_4_C_2_ expansion alleles in a healthy Italian population, identifying alleles associated with a healthy phenotype and thereby aiding in the clinical interpretation of results.

## 2 Materials and methods

### 2.1 Sample size evaluation

We conducted a one-sample proportions test with a continuity correction to estimate the 95% confidence interval (CI) for the population proportion of carriers of intermediate hexanucleotide repeat expansion (CHRE) (19–21). This CI was derived using the point estimate from samples with more than 24 and fewer than 30 repeats in the non-neurodegenerative European population. We extracted frequency data for each specified CHRE repeat range. It is important to note that calculating the standard error and margin of error is not feasible with 0 successes; hence, data for more than 30 CHRE ranges could not be included in this analysis. The inputs for the one-sample proportion tests were 607 carriers for the 2–23 CHRE range and 6 carriers for the 14–30 CHRE range (22–24).

Our findings indicate that, at a 95% confidence level, the actual frequency of carriers of intermediate HRE is likely between 0.003 and 0.02, with a margin of error (ME) of 0.01. Consequently, to achieve a 0.05 ME for surveying the distribution of 24–30 HRE carriers, we calculated the required sample size using Equation (1).


(1) $$n=\frac{1.96^{2}*P_{\text{exp}}\left(1-P_{\text{exp}}\right)}{{\mathrm{ME}}^{2}}$$

Equation (1)—Sample size estimation based on a proportion estimate at a 95% confidence level (25).

The required sample size for subjects to obtain a 0.05 ME is 1,430. Given the enrolled sample size of 967 subjects, we calculated the margin of error using the data from Equations (1) and (2) in Equation (3), obtaining a 0.063 ME.


(2) $$y=\frac{n*a}{\frac{b*n}{k}}$$

where *a* = enrolled sample size, *b* = total sampled subjects from literature, and *k* = number of HRE carriers in the range of interest (24–30) from b. Therefore, *y* is n proportional to *a*.


(3) $$\mathrm{ME}=\frac{1.96^{2}*P_{\text{exp}}\left(1-P_{\text{exp}}\right)}{y}$$

Equation (3) Expected proportion obtained from Equation (2) was used to compute the achieved ME with 967 subjects sampled.

### 2.2 Subjects enrollment

Nine hundred and sixty-seven elderly subjects (subjects over 70 years of age; median age 78, dev.st. 6.18; male-to-female ratio 1:1.34) were selected during the clinical neurologist’s routine activities. The inclusion criteria were being older than 70 years and having no family history of both ALS and FTD. Exclusion criteria encompassed the presence of any neurodegenerative disorders or *C9orf72*-associated phenotypes. Participants are not related to each other. Written informed consent was obtained from all participants. The study received approval from the Ethics Committee of all participating centers.

### 2.3 Proficiency test

To ensure the validity of the results, all participating institutes were required to perform proficiency testing (PT) before processing the samples. PT is crucial for quality assessment, especially in multicenter studies, as it benchmarks the performance of the participants. Each center involved in the study received five blind samples with known genotypes. The proficiency test was considered passed if a center accurately reported the sizes of the hexanucleotide G_4_C_2_ alleles. Upon successful completion, the center was granted access to the analytical phase of the study. If inconsistencies were observed, the center underwent specific training on the use of testing kits and interpretation software. Following this training, the center was required to repeat the PT using a different set of samples. Notably, among the blind samples was DNA with an indel variation in the hexanucleotide G_4_C_2_ allele (interruption in the G_4_C_2_ repeat).

### 2.4 Repeatability test

To confirm the stability of the AmplideXPCR/CE *C9orf72* Kit (Asuragen, Inc.) over time, each participating institution conducted a repeatability study using a subset of samples (n = 5). Every center involved in this study was supplied with five blind samples, each having a known genotype. These samples were tested at three different time points: T0 (initial testing), T1 (after 2 weeks), and T3 (after 4 weeks).

### 2.5 Genetic analysis

Genomic DNA was purified from peripheral blood samples using automated procedures (according to instrumental equipment available in IRCCS laboratories). The quality of the extracted DNA was checked by spectrophotometer analysis. Minimal quality parameters requested were: OD_260/280_ and OD_260/230_ ≥ 1.7; DNA [10–40 ng/μL].

Genomic DNA was typed using the AmplideXPCR/CE *C9orf72* Kit (Asuragen, Inc.). The assay consists of amplification using a three-primer G_4_C_2_-repeat primed (RP)-PCR configuration (21), followed by fragment sizing on a capillary electrophoresis (CE) instrument. PCR product (2 mL) mixed with formamide and ROX 1000 Size Ladder (Asuragen, Inc., Austin, TX) was run on a CE instrument (according to instrumental equipment available in IRCCS laboratories). The amplicons were detected according to the manufacturer’s protocols (26). The sizing of amplicons was performed by GeneMapper software according to the manufacturer’s protocols. Positive controls (*C9orf72* alleles with 2, 5, 8, and 10 repeats) and negative PCR controls were run in all experiments.

## 3 Results

To standardize and validate analytical methods, each participating institution was provided with the same lot of the commercially available AmplideXPCR/CE *C9orf72* Kit (Asuragen, Inc.). Additionally, they were required to conduct a proficiency test designed and distributed by Asuragen. As a result, 11 out of 13 centers successfully genotyped the blinded samples. Two centers, however, failed to correctly size a genotype that was homozygous for an expanded hexanucleotide G_4_C_2_ allele (>145 repeats). Consequently, these centers underwent specific training to improve their use of the AmplideXPCR/CE *C9orf72* Kit and to refine their allele sizing analyses through capillary electrophoresis. Following this intervention, the two centers were able to successfully retake and pass the proficiency test.

Once each center passed the proficiency test, a repeatability test was initiated, and all blind samples were typed at different times (T0, T1, and T3). The results demonstrated remarkable repeatability, with no variation observed in the serial measurements (repeatability coefficient = 0), thereby affirming the assay’s reliability in characterizing *C9orf72* alleles. Furthermore, one of the blind samples exhibited an indel variation in the hexanucleotide G_4_C_2_ allele (an interruption in the G_4_C_2_ repeat). All centers successfully genotyped this sample without any discrepancies in repeat sizing attributable to the indel variation, thereby providing the efficacy of the AmplideXPCR/CE *C9orf72* Kit in accurately sizing alleles, even in the presence of interruptions in the G_4_C_2_ repeat.

**Figure 2:**
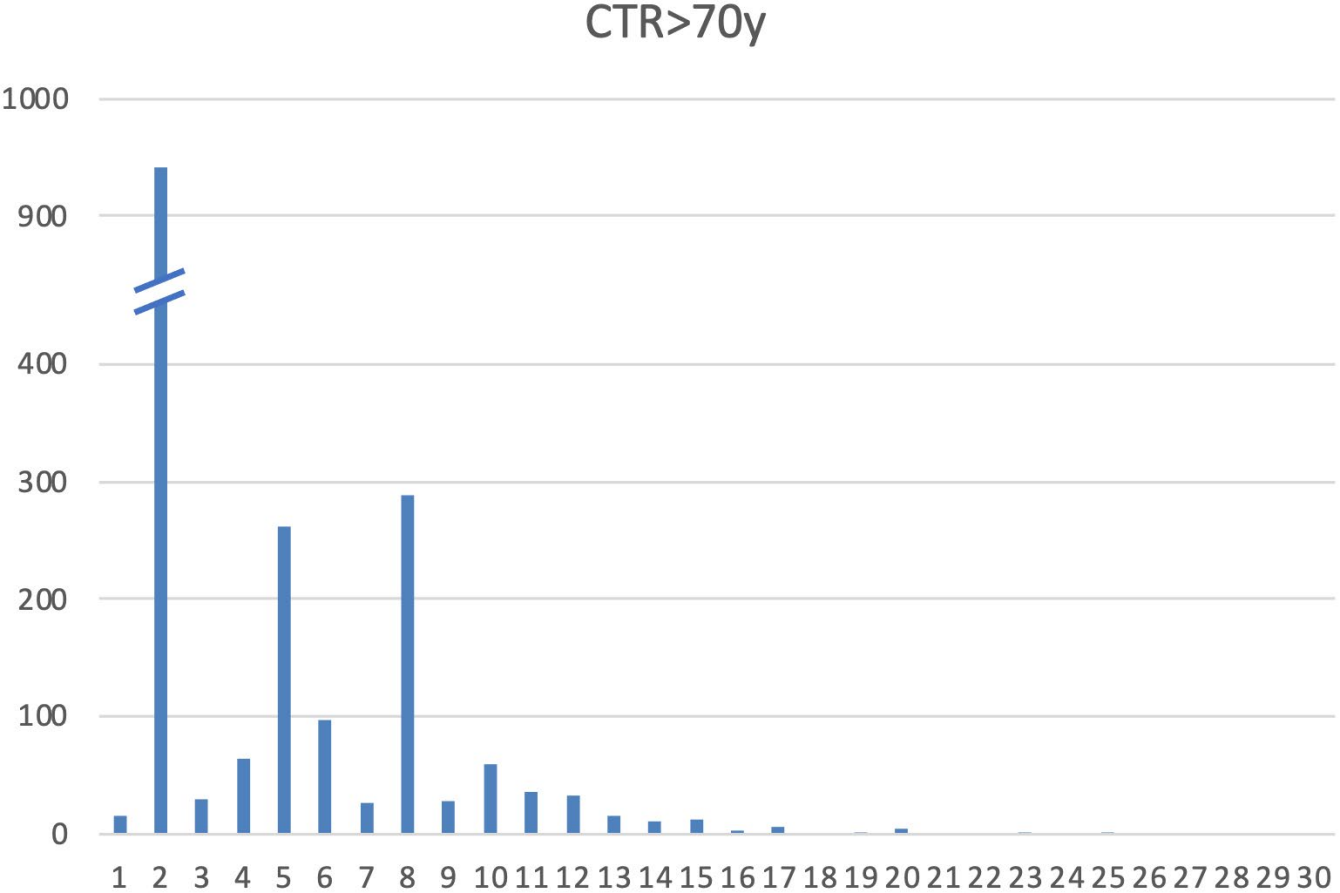
Allelic distribution of C9orf72 alleles in our cohort.

A total of 967 subjects were enrolled across 13 centers (as detailed in Supplementary Table S1—subjects per center). Alleles and genotype frequency were characterized in this cohort of healthy elderly Italian subjects. All subjects carried hexanucleotide G_4_C_2_ alleles with fewer than 25 repeats. There was no statistically significant variation in the distribution of repeat lengths between healthy samples analyzed by different centers (alpha = 0.05; KW χ^2^: 9.99, *p* = 0.616). The distribution of alleles is presented in Table 1 and Figure 2.

**Table 1:** Allelic distribution of C9orf72 alleles in our cohort and in other cohorts.

	Our cohort		UK controls (23)		England, Scotland, and Wales controls (31)		Multiethnic population (Europe, USA, Australia, Singapore, Japan) (32)		ADNI controls (23)		Finnish controls (27)		Ashkenazi Jewish (33)		Moroccan Jewish (33)	
Units	Number of alleles	Percentage	Number of alleles	Percentage	Number of alleles	Percentage	Number of alleles	Percentage	Number of alleles	Percentage	Number of alleles	Percentage	Number of alleles	Percentage	Number of alleles	Percentage
1	15	0.78%	n.a	n.a.	n.a.	n.a.	n.a.	n.a.	n.a.	n.a.	n.a	n.a.	n.a.	n.a.	n.a.	n.a.
2	941	48.66%	349	51.32%	1,386	42.08%	6,526	55.72%	283	51.83%	1,358	34.60%	797	66.53%	294	49.25%
3	29	1.50%	1	0.15%	2	0.06%	28	0.24%	3	0.55%			7	0.58%	4	0.67%
4	64	3.31%	14	2.06%	88	2.67%	250	2.13%	14	2.56%	77	1.96%	4	0.33%	6	1.01%
5	262	13.55%	112	16.47%	658	19.98%	1,576	13.46%	89	16.30%	1,012	25.78%	122	10.18%	82	13.74%
6	97	5.02%	35	5.15%	209	6.34%	696	5.94%	38	6.96%	210	5.35%	23	1.92%	30	5.03%
7	27	1.40%	14	2.06%	64	1.94%	340	2.90%	8	1.47%	118	3.01%	8	0.67%	2	0.34%
8	289	14.94%	80	11.76%	469	14.24%	1,373	11.72%	60	10.99%	665	16.94%	134	11.19%	107	17.92%
9	28	1.45%	7	1.03%	35	1.06%	134	1.14%	6	1.10%	23	0.59%	18	1.50%	5	0.84%
10	60	3.10%	23	3.38%	130	3.95%	277	2.36%	15	2.75%	203	5.17%	18	1.50%	25	4.19%
11	36	1.86%	14	2.06%	71	2.16%	179	1.53%	6	1.10%	41	1.04%	29	2.42%	20	3.35%
12	32	1.65%	4	0.59%	44	1.34%	124	1.06%	8	1.47%	38	0.97%	12	1.00%	4	0.67%
13	16	0.83%	9	1.32%	28	0.85%	80	0.68%	1	0.18%	27	0.69%	11	0.92%	0	0.00%
14	10	0.52%	5	0.74%	27	0.82%	67	0.57%	2	0.37%	38	0.97%	3	0.25%	10	1.68%
15	12	0.62%	4	0.59%	13	0.39%	37	0.32%	0	0.00%	20	0.51%	2	0.17%	1	0.17%
16	3	0.16%	2	0.29%	11	0.33%	26	0.22%	2	0.37%	16	0.41%	3	0.25%	0	0.00%
17	6	0.31%	0	0.00%	11	0.33%	0	0.00%	2	0.37%	11	0.28%	2	0.17%	3	0.50%
18	0	0.00%	0	0.00%	9	0.27%	0	0.00%	3	0.55%	10	0.25%	1	0.08%	0	0.00%
19	1	0.05%	0	0.00%	4	0.12%	0	0.00%	3	0.55%	6	0.15%	2	0.17%	1	0.17%
20	4	0.21%	1	0.15%	7	0.21%	0	0.00%	2	0.37%	8	0.20%	0	0.00%	0	0.00%
21	0	0.00%	0	0.00%	10	0.30%	0	0.00%	0	0.00%	4	0.10%	0	0.00%	0	0.00%
22	0	0.00%	0	0.00%	6	0.18%	0	0.00%	1	0.18%	12	0.31%	1	0.08%	0	0.00%
23	1	0.05%	0	0.00%	3	0.09%	0	0.00%	0	0.00%	7	0.18%	1	0.08%	1	0.17%
24	0	0.00%	0	0.00%	3	0.09%	0	0.00%	0	0.00%	6	0.15%	0	0.00%	0	0.00%
25	1	0.05%	0	0.00%	2	0.06%	0	0.00%	0	0.00%	8	0.20%	0	0.00%	2	0.34%
26	0	0.00%	1	0.15%	0	0.00%	0	0.00%	0	0.00%	4	0.10%	0	0.00%	0	0.00%
27	0	0.00%	0	0.00%	0	0.00%	0	0.00%	0	0.00%	0	0.00%	0	0.00%	0	0.00%
28	0	0.00%	0	0.00%	0	0.00%	0	0.00%	0	0.00%	0	0.00%	0	0.00%	0	0.00%
29	0	0.00%	0	0.00%	1	0.03%	0	0.00%	0	0.00%	0	0.00%	0	0.00%	0	0.00%
30	0	0.00%	0	0.00%	1	0.03%	0	0.00%	0	0.00%	0	0.00%	0	0.00%	0	0.00%
>30	0	0.00%	5	0.74%	2	0.06%	0	0.00%	0	0.00%	3	0.08%	0	0.00%	0	0.00%
Total	1934		680		3,294		11,713		546		3,925		1,198		597	

ADNI, Project MinE and Alzheimer’s Disease Neuroimaging Initiative (23).

All samples were characterized by alleles with fewer than 25 repeats. Specifically, 93.7% of samples had ≤10 repeats, 99.9% had ≤20 repeats, and 100% had ≤25 repeats (data shown in Table 1). The distribution of repeat lengths peaked at 2, 5, and 8 repeats, with frequencies of 48.7, 13.5, and 14.9%, respectively. The longest allele identified had 25 repeats, observed once, representing a frequency of 0.05%. Alleles with lengths ≥20 repeats had a frequency of 0.31%, which is slightly lower than the frequencies reported in other European populations (23, 27, 28) (Table 2).

**Table 2:** Comparison of frequency of ≥20 repeats in Caucasian populations (27).

Population	Allelic frequency of ≥20 repeats	Expansions in controls (n)	Reference
Italian (our study)	0.31	0	Present study
UK	0.42	11	Beck et al. (34)
Irish	0.41	0	Fahey et al. (35)
European/Asian/North American/Australian	0.52	1	Theuns et al. (32)
North American	0.38	0	Rutherford et al. (36)
Finnish	0.89	6	Kaivola et al. (27)

## 4 Discussion and conclusion

Since its first detection in 2011, clinical and research evidence has repeatedly suggested the introduction of the genetic test for *C9orf72* into clinical practice. In the present year, these suggestions have been definitively entered into ALS guidelines (29). Unfortunately, the extreme variability of the repeat in *C9orf72* makes it hard to standardize the analysis. First, the repeat expansion of *C9orf72* may vary in different tissues (30). Moreover, allelic frequency strongly varies among populations (3). It became evident that to promote the introduction of the genetic test for *C9orf72* into clinical practice, it was mandatory to standardize technologies and interpret results.

To standardize the genetic test for *C9orf72* and result interpretation, the RIN launched a national survey among laboratories performing G_4_C_2_ repeat evaluation for diagnostic purposes. As already observed in other countries (4, 17), the survey results highlighted interlaboratory heterogeneity for the cutoff between normal and pathological alleles and analytical methods. While the interpretation of clearly pathological and normal sizes has been recognized (respectively over 60 G_4_C_2_ repeats and under 8 G_4_C_2_ repeats), the exact definition of the cutoff for intermediate sizes remains challenging, mainly due to the absence of genetic data from the general and healthy population. In this scenario, the same intermediate alleles can be interpreted as normal or pathological in different laboratories, giving a dissimilar disease evaluation and risk prediction in genetic counseling. Given the known variability in allele distribution across different populations, the high frequency of *C9orf72* HRE alleles (23), and the interlaboratory survey results, the RIN performed a multicenter study to estimate the distribution of G_4_C_2_ expansion in healthy Italian elderlies (over the age of 70) and to promote the methods’ standardization across the institutes involved in the RIN network. Considering the quite complete penetrance of the *C9orf72*-associated phenotypes at 80 years of age (37), selecting a sample of neurologically healthy individuals over 70 years old seemed reasonable. Notably, factors such as family history, clinical presentation, and gender can slightly alter the median onset age, which typically ranges from 57 to 60 years (37). In the current study, a total of 967 elderly healthy subjects from the Italian population were typed to characterize the distribution of *C9orf72* alleles, defining the frequency of G_4_C_2_ repeat alleles in the Italian healthy population. All the analyzed samples had alleles with fewer than 25 repeats. Specifically, 93.7% of the samples had ≤10 repeats, 99.9% had ≤20 repeats, and all had ≤25 repeats (Table 1).

When comparing these findings with allelic distributions in other populations, similar peaks at 2, 5, and 8 repeats were noted (Table 1). Additionally, the frequency of alleles with ≥20 repeats in our cohort is coherent with the observed north-to-south gradient of allelic distribution in Europe. Specifically, Caucasian control populations exhibit frequencies ranging from 0.38 to 0.52 for alleles with ≥20 repeats (27), with an exceptionally high frequency of 0.89 in the Finnish population. The frequency of 0.31 observed in our Italian cohort aligns with the higher incidence of hexanucleotide repeat expansion (HRE) observed in the northern regions of Europe, as referenced in the study (28). This consistency not only reinforces the geographic variation in HRE incidence but also underscores the critical importance of stringent inclusion criteria for control subjects in genetic epidemiological studies, particularly those involving age-related diseases.

This research establishes the range of *C9orf72* alleles typically found in a healthy Italian population, specifically identifying alleles with up to 25 repeats as being associated with a normal phenotype. These findings, combined with observed alleles in patients, help delineate the thresholds for normal, intermediate, and pathological alleles within this population. This study supported a definition of normal allele ranges. The benefits of the study will be evident when the data are compared with *C9orf72* allele distribution in patients. The main limitation of the study is that it is not a case–control study, so we still cannot define intermediate and pathological thresholds. Nevertheless, the sample selection (elderly without any *C9orf72*-related phenotype) supports the exact definition of normal alleles, even in this extreme variable phenotypic presentation.

Furthermore, the study underscores the benefits of collaboration among institutes, particularly in the context of sharing genomic data to harmonize analytical methods and advance applied research. Initially, half of the Neuroscience Institutes were using in-house methods for sizing the hexanucleotide G_4_C_2_ expansion. By the study’s conclusion, all participating institutes had adopted a uniform, commercially available kit, facilitating a standardized national reference for interpreting the normal allele thresholds. This approach, exemplified by the RIN network’s model, is also a concept study to promote the achievements expected from the entry into force of the *in vitro* Diagnostic Regulation (EU) 2017/746 (IVDR) at the European level. Furthermore, this achievement can largely contribute to the European Network for Rare Diseases (ERN) offering a homogenous assay to test *C9orf72* in the European Union; the ENCALS (European Network to Cure ALS) can equally consider the positive result of homogenization in technology obtained with the study as a referral for further initiatives aiming for broad consensus on analyzing G_4_C_2_ repeat expansion in *C9orf72.*

## Data availability statement

The datasets presented in this article are not readily available because of ethical and privacy restrictions. Requests to access the datasets should be directed to the corresponding author.

## Ethics statement

The studies involving humans were approved by Ethical Committee of the coordinating center (IRCCS Fondazione Santa Lucia): Prot. CE/PROG.936. The studies were conducted in accordance with the local legislation and institutional requirements. The participants provided their written informed consent to participate in this study.

## Author contributions

EG: Conceptualization, Methodology, Project administration, Visualization, Writing – review & editing, Writing – original draft. PM: Investigation, Resources, Supervision, Validation, Writing – review & editing. RG: Investigation, Resources, Supervision, Validation, Writing – review & editing. NT: Investigation, Resources, Supervision, Validation, Writing – review & editing. GR: Investigation, Resources, Supervision, Validation, Writing – review & editing. CF: Investigation, Resources, Supervision, Validation, Writing – review & editing. FT: Investigation, Resources, Supervision, Validation, Writing – review & editing. FE: Investigation, Resources, Supervision, Validation, Writing – review & editing. SC: Investigation, Resources, Supervision, Validation, Writing – review & editing. BN: Investigation, Resources, Supervision, Validation, Writing – review & editing. RoM: Investigation, Resources, Supervision, Validation, Writing – review & editing. RC: Data curation, Formal analysis, Investigation, Writing – review & editing. LP: Data curation, Formal analysis, Investigation, Writing – review & editing. FS: Data curation, Formal analysis, Investigation, Writing – review & editing. SZ: Data curation, Formal analysis, Investigation, Writing – original draft, Writing – review & editing. CPe: Data curation, Formal analysis, Investigation, Writing – original draft, Writing – review & editing. IP: Data curation, Formal analysis, Investigation, Writing – review & editing. CG: Data curation, Formal analysis, Investigation, Writing – review & editing. EN: Data curation, Formal analysis, Investigation, Writing – review & editing. PO: Data curation, Formal analysis, Investigation, Writing – review & editing. FG: Data curation, Formal analysis, Investigation, Writing – review & editing. CPo: Data curation, Formal analysis, Investigation, Writing – review & editing. RN: Data curation, Formal analysis, Investigation, Writing – review & editing. LB: Data curation, Formal analysis, Investigation, Writing – review & editing. SP: Data curation, Formal analysis, Investigation, Writing – review & editing. AR: Data curation, Formal analysis, Investigation, Writing – review & editing. MRi: Data curation, Formal analysis, Investigation, Writing – review & editing. GF: Data curation, Formal analysis, Investigation, Writing – review & editing. SM: Data curation, Formal analysis, Investigation, Writing – review & editing. MS: Data curation, Formal analysis, Investigation, Writing – review & editing. SL: Data curation, Formal analysis, Investigation, Writing – review & editing. TD: Data curation, Formal analysis, Investigation, Writing – review & editing. PC: Data curation, Formal analysis, Investigation, Writing – review & editing. ES: Data curation, Formal analysis, Investigation, Writing – review & editing. SB: Data curation, Formal analysis, Investigation, Writing – review & editing. AI: Data curation, Formal analysis, Investigation, Writing – review & editing. AA: Data curation, Formal analysis, Investigation, Writing – review & editing. FT: Funding acquisition, Project administration, Supervision, Writing – review & editing. RL: Funding acquisition, Project administration, Supervision, Writing – review & editing. CC: Funding acquisition, Project administration, Supervision, Writing – review & editing. SG: Data curation, Methodology, Supervision, Writing – review & editing. EV: Data curation, Methodology, Supervision, Writing – review & editing. VS: Conceptualization, Supervision, Visualization, Writing – review & editing. FA: Investigation, Resources, Supervision, Validation, Writing – review & editing.
